# Autoimmunity and Asbestos Exposure

**DOI:** 10.1155/2014/782045

**Published:** 2014-04-29

**Authors:** Jean C. Pfau, Kinta M. Serve, Curtis W. Noonan

**Affiliations:** ^1^Department of Biological Sciences, Idaho State University, 921 South 8th Avenue, Stop 8007, Pocatello, ID 83209, USA; ^2^Center for Environmental Health Sciences, University of Montana, Missoula, MT 59812, USA

## Abstract

Despite a body of evidence supporting an association between asbestos exposure and autoantibodies indicative of systemic autoimmunity, such as antinuclear antibodies (ANA), a strong epidemiological link has never been made to specific autoimmune diseases. This is in contrast with another silicate dust, crystalline silica, for which there is considerable evidence linking exposure to diseases such as systemic lupus erythematosus, systemic sclerosis, and rheumatoid arthritis. Instead, the asbestos literature is heavily focused on cancer, including mesothelioma and pulmonary carcinoma. Possible contributing factors to the absence of a stronger epidemiological association between asbestos and autoimmune disease include (a) a lack of statistical power due to relatively small or diffuse exposure cohorts, (b) exposure misclassification, (c) latency of clinical disease, (d) mild or subclinical entities that remain undetected or masked by other pathologies, or (e) effects that are specific to certain fiber types, so that analyses on mixed exposures do not reach statistical significance. This review summarizes epidemiological, animal model, and *in vitro* data related to asbestos exposures and autoimmunity. These combined data help build toward a better understanding of the fiber-associated factors contributing to immune dysfunction that may raise the risk of autoimmunity and the possible contribution to asbestos-related pulmonary disease.

## 1. Introduction

**Figure 1:**
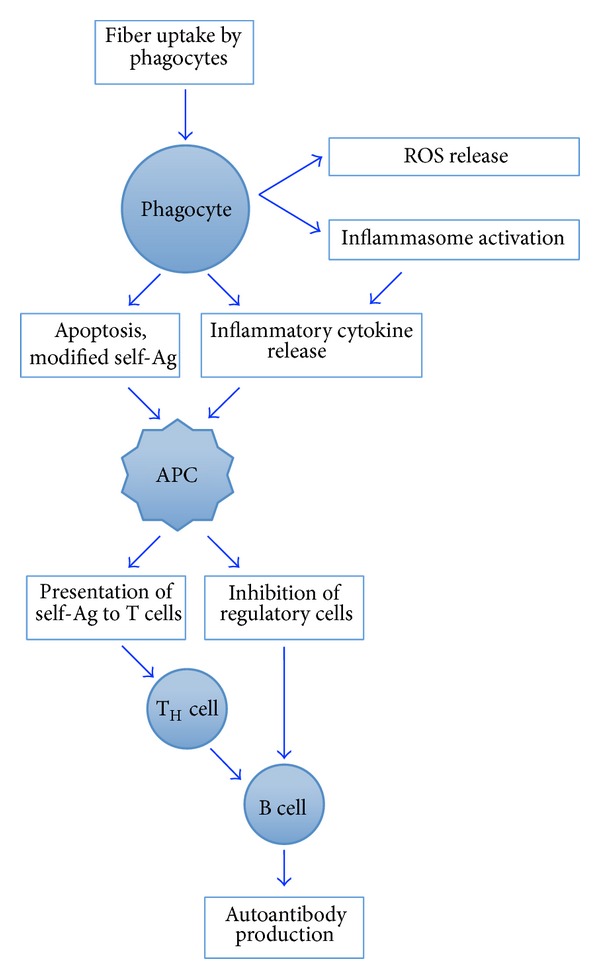
Schematic of possible players in the immune dysfunction by mineral fibers. These are putative mechanisms only. More details on mode of action are covered in excellent reviews mentioned in the text [2, 3, 16, 17].

Autoimmune disease is the clinical manifestation of abnormalities in immune regulation that lead to tissue damage by self-reactive lymphocytes and autoantibodies, resulting in debilitating symptoms and death when vital organs are affected. The cause(s) of most autoimmune diseases remain uncertain, although environmental factors are strongly indicated through studies in animal models [1]. Systemic autoimmune diseases (SAID) including systemic lupus erythematosus (SLE), systemic sclerosis (SSc), and rheumatoid arthritis (RA) appear to have complex etiologies with gene-environment interactions [2]. Silicate dusts, including crystalline silica and asbestos, increase production of autoantibodies, possibly through the production of excess cellular debris in the context of a highly inflammatory environment [2–4]. However, the exact mechanisms (apoptotic pathways, cytokine patterns, and redox regulation) by which exposure to silicate dusts drives autoimmune responses are not clearly elucidated, and it is not known whether this is a universal response to inhaled mineral dusts (Figure 1).

Exposure to crystalline silica leads to increased antinuclear autoantibodies (ANA) in both mice and humans and increases the risk of SLE, RA, and SSc [2, 5, 6]. While this association with silica exposure is widely accepted, asbestos exposure has not yet been strongly linked with any particular autoimmune or connective tissue disorder. Nevertheless, there are reports of immune abnormalities and humoral indices consistent with autoimmune mechanisms, including a variety of autoantibodies such as ANA and rheumatoid factor (RF) (detailed below). Several factors could be contributing to the inability to associate asbestos with SAID epidemiologically, including (a) a lack of statistical power due to relatively small or diffuse exposure cohorts, (b) exposure assessment issues, (c) the latency of the clinical disease, and (d) mild clinical or subclinical entities that remain undetected or masked by other pathologies. In addition, a key factor may center around the definition of asbestos in these studies.

The term “asbestos” is generally regarded as broadly descriptive of mineral fibers used commercially due to their durability and heat resistance. Specifically, they are defined as being long and thin (having an aspect ratio greater than 3 : 1), and falling into categories of either “serpentine” (chrysotile) or “amphibole” (tremolite, amosite, crocidolite, actinolite, and anthophyllite) [7]. As a group, asbestos has been classified as a carcinogen and is known to cause a pulmonary fibrotic disease called “asbestosis.” Despite this generalization, all of these fiber types have distinct physicochemical properties (shape, durability in physiological fluids, surface chemistry, and aerodynamic properties), making the term “asbestos” mineralogically imprecise [7] (see Table 1). In 2010, the U.S. Environmental Protection Agency (EPA) and the National Institute of Environmental Health Science (NIEHS) jointly convened a workshop to invite experts from all areas of asbestos research and toxicology in order to address these issues of nomenclature and dosage and to better understand the modes of action (MOA) behind asbestos-induced health effects [8, 9]. Part of the impetus behind this effort was the awareness of severe health problems that have occurred as a result of exposure to mineral fibers in contaminated vermiculite mined just outside of Libby, Montana. Much of the fibrous material, including winchite and richterite, did not fall into the definition above, despite containing long, thin “asbestiform” mineral fibers. Since then, another mineral fiber in the zeolite family called erionite has been shown to be highly carcinogenic and causing pulmonary diseases similar to those seen with asbestos [10–12]. In addition, over the last few decades the manufacture and use of nanomaterials called “nanotubes” and “nanowires” have dramatically increased, leading to health concerns due to similarities to asbestos [13, 14]. The imprecision in asbestiform fiber classification means that it is impossible to generalize about associated health outcomes, since one type of fiber might have very different health effects based on its ability to be inhaled into deep regions of the lung, surface properties that affect the interaction with cells, and the amount to which it is cleared by the innate immune system [15]. Until recently, much of the literature on the health effects of mineral fibers was focused on occupational exposures to asbestos. Industrial hygiene and work records data available for occupationally exposed cohorts enable quantitative exposure assessment methods, but such methods are typically focused on one fiber type such as chrysotile. Such analyses do not allow for the possibility of the mixed fiber exposures or account for the potentially disparate health outcomes associated with different fiber exposures. Occupational exposures also are comprised primarily of men, but autoimmune diseases are often more common among women. It is very possible, therefore, that the lack of epidemiological evidence in support of an association between asbestos exposure and autoimmunity is because the studies exploring this issue have been focused on different (or mixed) mineral fiber types in occupational, rather than general, populations.

This review describes the evidence for induction of autoantibodies following asbestos exposure, the enigmatic epidemiological data regarding an association with SAID, and then explores hypotheses that might help explain the discord between the two types of data. Finally, we present emerging data that support the presence of tissue specific autoantibodies that may play a critical role in the severity or progression of asbestos-associated pulmonary disease. Identification of weaknesses and limitations within available epidemiological data are important to help strengthen design of future studies since exposures to mineral fibers will continue to present public health challenges long into the future.

## 2. Asbestos Exposure and Autoantibodies

A small number of epidemiological studies explore an association between asbestos exposure and autoantibody responses (see Table 2). Cross-sectional associations between humoral responses, including rheumatoid factor (RF) and ANA, among asbestos workers were initially reported in 1965 [18]. Subsequent reports described increased ANA frequency with asbestos exposure, as well as increased serum IgG/IgA and immune complexes [19–25]. A few studies indicate no increase in ANA [25–27]. Most recently, subjects exposed to the Libby, MT, amphibole were shown to have elevated frequency and titers of ANA compared to a reference population [20]. Among the autoantibodies detected were those that target common SLE autoantigens, including dsDNA, SSA/Ro52, and ribonuclear proteins (RNP) [20, 28]. An increased frequency of positive RF tests among asbestos workers compared to the general population has been reported in several studies [21, 23, 29], while others reported no association [20, 24, 27]. It is highly likely that differences in serum dilutions and technical approaches can explain some of these differences. An early, sensitive detection marker for RA, antibodies to cyclic citrullinated proteins (anti-CCP), was not elevated in a subset of the Libby amphibole-exposed population [28].

Exposure to amphibole asbestos increases the frequency of positive ANA tests in nonautoimmune prone mice and rats [39–41] (Table 3). Mice exposed to amphibole asbestos (tremolite) exhibited immune complex deposition in the kidneys and mild glomerular changes suggestive of lupus nephritis [40]. The amphibole initially obtained by the U.S. Geological Survey (USGS) from the Libby mine site has been described as “6-Mix” because it was collected from six different sites, combined and characterized [31]. It is a combination of amphiboles including winchite, richterite, tremolite, and amosite and is very likely similar to the material to which the miners and townspeople were exposed over decades of mining the asbestos-contaminated vermiculite [31]. This material (LA (Libby amphibole)) has also been shown to induce ANA in intratracheally exposed mice [39] and rats [41, 42]. In the rat studies, a more pure sample of amphibole asbestos (amosite) was also shown to induce ANA in the rats [41, 42].

The combined human and animal data suggest that there are autoimmune responses associated with asbestos exposure that include autoantibodies characteristic of SAID, particularly SLE. Although autoantibodies are often present prior to onset of clinical disease [44], it might be expected that epidemiological data would report SAID in asbestos-exposed populations.

## 3. Systemic Autoimmune Disease (SAID) and Asbestos

Like the serological studies, previous epidemiological assessments of SAID in asbestos-exposed cohorts were fairly small studies and tended to suffer from problems with exposure assessment [45]. Rheumatoid arthritis has been the SAID most frequently associated with asbestos exposure [46–48]. Other SAIDs are extremely rare with prevalence estimates ranging from 4 to 24 per 100,000 populations, resulting in challenges to statistical power for studies conducted among relatively small asbestos-exposed populations. Nevertheless, one study described an increased risk for SSc deaths among persons having occupations with likely exposure to asbestos [49]. A recent case-control study of self-reported SLE or SSc patients nested within a medically screened general population cohort in Libby, MT, showed associations for both diseases with amphibole exposure [47].

An association with ANCA-associated vasculitis has been described in two studies of asbestos exposures [50, 51] but was not found in at least one study despite an association with silica exposure [52]. Because the interstitial pneumonia that is common in this form of vasculitis can be mistaken for asbestosis, this link may simply be overlooked. Several studies also report an association between asbestos exposure and periaortitis and retroperitoneal fibrosis, both of which are considered autoimmune diseases [53–57]. This pathology is of interest due to the fiber burden of tissues in this area of the body following asbestos exposure [58].

Two groups have examined symptoms of systemic autoimmune disease in animal models after asbestos exposure. In addition to inducing ANA in C57BL/6 mice, tremolite was shown to increase immune complex deposition in the kidneys of exposed mice [40]. In that study, the autoantigen targets for the ANA included dsDNA, Ro52, and RNP, which are common in human SLE. However, neither proteinuria nor overt kidney disease was significantly increased over the experimental period. In rats, despite production of ANA after exposure to Libby amphibole or amosite, there was no evidence of exacerbated disease in a model of induced RA [42]. These fibers increased proteinuria in the rats but did not increase immune complex deposition or kidney pathology [41]. Therefore, to our knowledge there have been no studies that clearly demonstrate induction or exacerbation of SAID by mineral fibers in animal models.

Taken together, these studies make a compelling, but not definitive, case for an association between “asbestos” and immune dysfunction relevant to autoimmunity. Many of the human studies suffer from technical issues such as small study sizes, predominantly male occupational cohorts and limited exposure data. For example, one study indicated no association of positive ANA tests with asbestos exposure, but that study only consisted of 25 asbestos workers, and there was no clear definition of the type of asbestos [27]. A small study of 66 anthophyllite miners showed no induction of ANA, but the method of measurement is unclear [34]. As indicated in Table 2, most studies indicate the occupation but not the fiber types. Incidences where persons are exposed to pure chrysotile or amphibole are rare, so most of these studies represent mixed exposures of unknown proportions. However, a recent review reported on the perceived strength of the literature support for the association of asbestos exposure with autoimmunity, and the strongest data was shown to be in studies of tremolite, an amphibole asbestos, or mixtures with heavy amphibole content [59]. This therefore raises the issue of the different mineralogy of these fibers and whether they have similar effects in immune dysfunction.

## 4. Hypotheses Regarding the Discordant and Inconsistent Results

There are several possible explanations for the lack of strong epidemiological data supporting a link between asbestos and autoimmune disease. First, asbestos exposure cohorts tend to be small and composed predominantly of males. With the possible exception of rheumatoid arthritis, SAIDs are rare with estimated prevalence in the U.S. general population of 24 per 100,000 for SLE, 5 per 100,000 for polymyositis/dermatomyositis, and 5 per 100,000 for systemic sclerosis [45]. Prospective epidemiological studies of rare disease require large cohorts followed for extended periods of time. Case-control studies can overcome some of these challenges, but asbestos is a relatively rare exposure and difficult to adequately assess retrospectively in the general population. Thus, epidemiological studies of asbestos exposure and risk of SAID often have limited statistical power even when evaluating associations with large effects sizes. SAIDs, including rheumatoid arthritis, are also more prevalent among women who account for 67% to 92% of SAID prevalence [45]. By contrast, occupational asbestos-exposed cohorts are predominantly male. Several studies have evaluated respiratory disease outcomes among women exposed to take-home asbestos from their male occupationally exposed spouses [60], but epidemiological studies of autoimmune disease outcomes among exposed women have rarely been conducted [61].

**Figure 2:**
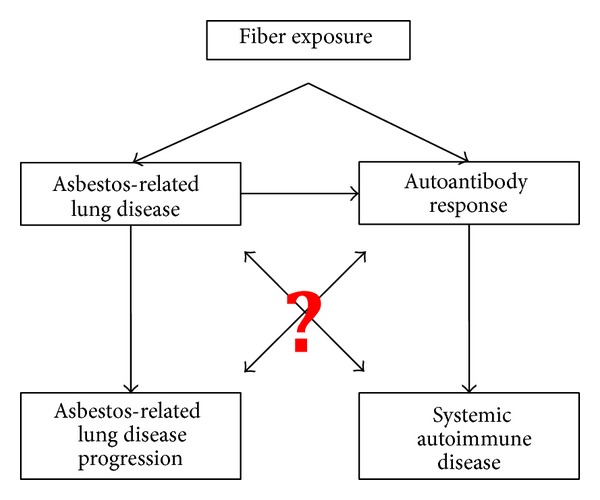
Proposed relationships between asbestos exposure, autoimmunity, and fibrotic lung disease progression. Data (as mentioned in the text) support the connections indicated, but questions remain regarding (a) the types of fibers that are responsible and (b) the etiological and mechanistic bases for the outcomes.

Second, autoantibodies may not contribute significantly to pathology and may be the result of chronic damage and inflammation associated with asbestos-related pleural disease. The long, but uncertain and variable, latency of autoimmune changes further limits the epidemiological approaches that can be employed to elucidate these relationships (Figure 2). Longitudinal studies are required to disentangle this potential issue of reverse causality. To date only one study specifically addressed the temporal nature of the asbestos/autoimmune/lung pathology complex by following a cohort of workers in an asbestos plant [22, 38]. The baseline study demonstrated the presence of an increased frequency of ANA in this cohort, along with radiological changes in the workers' lungs [22]. The follow-up study demonstrated that subjects with ANA were more likely to develop radiologic abnormalities than subjects who were ANA negative [38]. These results, along with the knowledge that, in general, autoantibodies occur quite early in SLE patients, before clinical onset [62], argue against the hypothesis that autoantibodies associated with asbestos exposure occur after lung disease is already apparent clinically. A general population cohort that has been environmentally and occupationally exposed to amphibole asbestos is currently being followed to further examine the temporal relationship between autoantibodies and lung disease [63].

Third, limited attention to fiber type in epidemiological studies may result in fiber-specific exposure misclassification. Bernstein et al. have shown that chrysotile is less biopersistent than amphibole [64], likely leading to a shorter time in contact with immune system. It might take extended periods in the presence of fibers to create the local environment of accumulating cell debris combined with a combination of cytokines that stimulate self-reactive lymphocytes [2, 3]. While the definition of asbestos includes both families, amphiboles and chrysotile, the fibers are clearly distinct morphologically and have unique physicochemical properties [65]. Common health outcomes of asbestos inhalation include lung carcinoma, interstitial fibrosis (asbestosis), pleural scarring, and mesothelioma, but there is no clear distinction regarding the toxicology of individual fiber types [15]. There is, however, quite a bit of evidence that amphibole asbestos seems to be more pathogenic, especially in terms of scarring of the lung parenchyma and pleura and possibly cancers as well [64, 66]. Because two recent studies from the Libby, MT cohort have indicated an association between the presence of autoantibodies and more severe disease, this makes it even more important to determine the immunotoxicological properties of specific forms of asbestos [20, 67]. There is a great deal of disagreement in the literature regarding the relative impact of different fiber types on cancer, pulmonary fibrosis, pleural disease, and immune parameters. A study in rats showed that chrysotile (Sumas Mountain) induced worse lung fibrosis compared to Libby amphibole and tremolite [68]. Dosages were made comparable by elutriation for rat-respirable fibers and by comparing exposure by mass, length, and aspect ratio. Other studies have reported significantly worse pulmonary and pleural fibrosis among amphibole-exposed subjects compared to chrysotile [64]. Therefore, there is clearly not a simple relationship between fiber type and specific disease end points.

In addition, there is evidence that chrysotile may induce long-term immunosuppressive effects among lymphocytes subsets of mesothelioma patients, leading to susceptibility to cancer but not autoimmune responses [35, 69, 70]. Comparisons with silica support the hypothesis that chrysotile does not induce the chronic immune activation/inflammation seen with silica that seems to drive the elevated risk for autoimmune diseases among silica exposed subjects [70]. This hypothesis is also supported by the work by a Japanese group [30, 71] that has shown immunosuppression in chrysotile exposed cells* in vitro* and* ex vivo*. Particular cells affected included cytotoxic T cells and NK cells, which were both suppressed by chrysotile, but not crocidolite, an amphibole [30]. The section below further reviews the literature comparing immunological parameters affected by amphibole versus chrysotile asbestos.

## 5. Amphibole versus Chrysotile: Autoimmunity

A recent* in vitro* comparison of the effects of Libby amphibole (6-Mix) and chrysotile on THP-1 monocytic cells and epithelial cells showed differential effects on inflammation/inflammasome activation [72]. Although both fibers activated the NLRP-3 inflammasome, amphibole appeared to do so via reactive oxygen species, while the response with chrysotile may have been mediated through lysosomal rupture. Therefore, these fibers induce very early innate immune responses for which these differences could greatly impact downstream consequences.

C57BL/6 mice were used to compare exposure to amphibole with chrysotile asbestos in terms of autoimmune responses [39]. While Libby amphibole induced ANA in a significantly higher proportion of the mice compared to controls (saline), chrysotile did not [39]. In addition, serum cytokines profiles in the mice exposed to amphibole were quantitatively and qualitatively different than in the chrysotile-exposed mice, including a dramatically elevated mean concentration of serum IL-17. The serum cytokines for chrysotile exhibited a T_H_1 profile, suggestive of mild chronic inflammation, with no elevation of T_H_2 cytokines or of IL-17. However, the results in the amphibole mice clearly suggest a T_H_17 response. The T_H_17 response is characterized by high levels of IL-17, triggered or maintained by other cytokines such as IL-6, IL-23, and TGF-beta [73]. T_H_17 responses have been implicated in a variety of diseases, including RA, SSc, and SLE [74–76]. In the above experiments, dosages were on a mass basis [39]. Therefore, due to differences in length and width of the different fiber types, mice were exposed to different numbers of fibers and total fiber surface area, dependent on fiber type. Since the surface area per mass of chrysotile is higher than for the amphiboles used, one might expect the effects of chrysotile to be greater, based on studies showing that surface area may be a critical factor in the pathogenicity of fibers [15, 77]. However, the results suggest the opposite: in these mice, chrysotile exposure is not associated with autoimmune responses. The only mechanistic hypothesis that emerged from this study seemed to support the idea of an immunosuppressive effect of chrysotile; in that an increased frequency of B suppressor cells was found in both the spleen and lungs of the chrysotile-exposed mice, but not amphibole [39]. Because the evidence suggests a very different kind of immune dysfunction induced by different fiber types, it is critical to examine the possible mechanisms by which autoantibodies might impact disease processes in asbestos-exposed patients.

## 6. Targets of Autoantibodies and Mechanisms of Disease

It has been suggested that identification of the specific targets of the autoantibodies might help in the development of hypotheses regarding mechanism of action, as well as diagnosis and progression of SAID [28]. Few studies have attempted to identify specific targets for asbestos-induced ANA, but one commonality has been the presence of anti-dsDNA in both mice and humans [20, 40, 78], but not rats [41]. Antibodies to neutrophils (ANCA) have been associated with silica and asbestos exposure [51, 79], but the asbestos exposure data came from an occupational exposure questionnaire, so the exposures likely included mixed chrysotile and amphibole. Pfau et al. did not find an association with ANCA in their amphibole-exposed cohort [20]. Recently, extractable nuclear antigen (ENA) specificities were reported for amphibole and chrysotile-exposed mice [39], but the number of ENA positive animals was too low to show any statistically significant differences. Interestingly, however, the Libby amphibole exposed mice showed a high frequency of anti-Jo-1 antibodies, similar to the rat study that showed significantly elevated positive tests for anti-Jo-1 with amphibole exposure [41]. Jo-1 autoantibodies have been shown to be associated with pulmonary disease [80], but the mechanism is not known.

Excellent reviews have explored the immunological effects of asbestos and attempted to link the various pathologies via a unified immune dysregulation [16, 17]. One of the recurring ideas regarding silica and asbestos immunotoxicology is that there are two events that converge to perpetuate autoimmune responses. The first is silicate-induced apoptosis, particularly of phagocytic cells, leading to accumulation of cellular debris. The second event is immune activation via “adjuvant” or inflammasome-activating effects, which drive antigen presentation in an environment that is no longer tolerized to self-material (Figure 1). Recent studies describe activation of inflammasomes by asbestos, driving proinflammatory effects such as IL-1*β* secretion [81, 82]. The inflammasome cascade activation, which can trigger a wide range of effects, may help explain the extremely diverse effects of asbestos in surface markers and cytokines that have been reported over the years [70, 83–86]. Despite the appeal of this 2-hit theory to link asbestos pathologies, the literature so far supports association, but not necessarily causation [87, 88]. However, there is the one study recently suggesting differential inflammasome activation by chrysotile and amphibole [72], which supports the idea that a key early trigger involves the inflammasome. This study demonstrated that although caspase cascade, oxidative stress, and the NLRP3 inflammasome were activated by both fibers, there were important differences in the specific pathways that were activated.

Interestingly, the murine SLE-like disease induced in mice by Libby amphibole was characterized by the production of autoantibodies to dsDNA and Ro52, similar to what was seen in the Libby asbestos human exposures [20, 28]. Such studies may be critical to discovery of mechanism of action. For example, it has been postulated that autoantigens become antigenic due to proteolytic degradation or apoptotic processes [2, 3]. During cell stress or death, Ro52 undergoes intracellular translocation and accumulates in apoptotic blebs during programmed cell death induced by a variety of oxidant challenges including asbestos [4, 89]. One study demonstrated that autoantibodies from asbestos-exposed mice bind to apoptotic blebs in which Ro52 had accumulated [88]. Ro52 has been identified as an E3 ubiquitin ligase [90], so it is possible that exposure to fibers causes upregulation of Ro52 expression, protein misfolding, and/or altered ubiquitination by Ro52 (including self-ubiquitination of Ro52 itself) and ineffective proteasomal degradation. Alteration or poor removal of target proteins could support such proteins becoming antigenic. One hypothesis, therefore, regarding the differences between immune dysfunction with amphibole and chrysotile relates to increased biopersistence of amphibole compared to chrysotile, so that long-term exposure to the fibers leads to accumulation of antigenic cell debris in an inflammatory environment, supporting the development of highly activated APCs that could then trigger autoreactive T and B cells. Alternatively, since both amphibole and chrysotile asbestos can cause oxidative stress and cell death in macrophages and mesothelial cells [91, 92], the mechanism of cell activation and apoptosis may be different [72], leading to different pathways of protein degradation.

Much more work is clearly needed to understand the mechanistic etiologies of the differential immune dysfunction by chrysotile and amphibole. The importance of this on-going discovery is illustrated in an examination of the relationship between autoantibodies and pulmonary disease, which strongly suggests exacerbation of disease.

## 7. Relationship between Autoimmunity and Pulmonary Disease

Several of the studies reporting ANA following asbestos exposure also indicated that having a positive ANA test was associated with either more severe or more rapid progression of lung disease (see Table 2, Figure 2) [20, 33, 38, 93]. The significance of this requires careful scrutiny, since it is possible that this association exists simply because high levels of exposure to asbestos may lead to both lung disease and autoantibodies, but that the latter two are not causally related. At least one study has shown no association between the presence of autoantibodies and radiological changes [37]. As mentioned above, it could also be that the autoantibodies follow the lung disease due to tissue damage, although the longitudinal studies by Tamura et al. argue against this since the autoantibodies were present prior to lung disease in many cases [22, 38]. Others have concluded that the lack of autoantibodies in other chronic pulmonary diseases also argue against the idea of the autoantibodies being only secondary to pulmonary disease [33, 93]. There are some clues among the various studies that might help elucidate whether there is an autoimmune component driving severity or progression of asbestos-related pulmonary disease. In the Tamura studies, where an association existed between increased ANA frequency with pulmonary lesions among asbestos-exposed workers, the association was only significant for interstitial, not pleural, lesions [22]. Although not clearly indicated, these were occupational exposures that were likely primarily chrysotile or a mixture of fibers. Another study, however, suggested that ANA in a tremolite (amphibole) exposed cohort were associated with pleural abnormalities [24]. Among former and current Libby, Montana residents, radiographic abnormalities were seen in 18% of the total population; however, among those with suspected SAID, nearly twice as many (35%) had radiographic abnormalities [94]. A follow-up study of this cohort revealed that LA-exposed individuals testing positive for ANAs were nearly 3.55 times more likely to have pleural or interstitial abnormalities than were those testing negative (*P* = 0.004) [67]. In the Libby cohort studies to date, the analyses were done simply for radiographic abnormalities, whether pleural or interstitial, primarily due to the fact that the vast majority of Libby subjects exhibit pleural disease, making analysis of interstitial disease alone very difficult [94]. Thus, these studies suggest the possibility that studies of cohorts (or animal models) exposed to pure chrysotile or amphibole asbestos might reveal very different autoantibody profiles that contribute to different forms of disease.

A possible role of autoantibodies to fibroblasts, endothelial, and epithelial cells in vascular and fibrotic disorders is receiving increasing attention as the evidence of autoantibody pathogenicity expands. Autoantibodies to endothelial cells have been implicated in vasculitis [95], SSc [96], and SLE [97]. Antifibroblast antibodies (AFA) are also considered a possible factor in pathogenesis of SSc [98–100]. However, data on the role of autoantibodies in fibrotic disease is emerging slowly, due to difficulties in assigning etiology in these complex disease processes (Figure 2). Autoantibodies are thought to contribute to fibrosis by activating target cells to produce profibrotic or proinflammatory cytokines [98], to secrete extracellular matrix proteins such as collagen I [43, 101], or by activating profibrotic cell signaling pathways [102]. Antifibroblast antibodies have been demonstrated in amphibole-exposed mice, and these AFA activate a phenotype change to myofibroblasts in mouse primary lung fibroblasts [43]. Based on the phosphorylation of PDGF-R alpha following treatment of these cells with serum antibodies from these mice, it was postulated that this receptor could be one of the targets for the autoantibodies [43]. In fact, AFA have been shown to bind to PDGF-R in SSc subjects, inducing profibrotic signaling [102]. Recently, mesothelial cell autoantibodies (MCAA) were found in sera of Libby amphibole-exposed subjects, and there was a positive and significant correlation between MCAA presence and pleural, but not interstitial, disease [67]. MCAA bind to the surface of pleural mesothelial cells (Met5A) and induce the production of collagen matrix in the absence of mesothelial-mesenchymal transition [101]. Thus, AFA and MCAA are found in the serum of amphibole-exposed mice and humans, respectively, and potentially contribute directly to the fibrotic disease process.

## 8. Conclusions

The limited number of epidemiological studies exploring a causal association between asbestos exposure and autoimmune disease makes it difficult to draw conclusions. First, as with most studies of asbestos, the observations of immune dysfunction described above are focused primarily on male, occupationally exposed populations. This could be a limitation when evaluating clinical outcomes such as autoimmune diseases that are more prevalent among women. Second, many studies are retrospective, introducing limitations in terms of exposure assessment and in clarifying the temporal relationship between exposure, autoimmune response, and pulmonary manifestations of disease. It is possible that asbestos exposure is associated with autoimmune disease processes that are not yet clinically recognized. Asbestos exposure in general, or exposure to specific fibers, may be associated with distinct autoimmune pathologies and serological responses that fall outside standard diagnostic criteria. This presents a unique challenge for epidemiological studies that often rely on medical records, physician assessment, death records, or other documentation to assess clinical endpoints. Nevertheless, the data summarized here provide compelling evidence of an association between asbestos exposure and autoimmunity, including a possible contribution of autoantibodies to the fibrotic disease process. It will be critical for future studies to carefully examine immune dysfunction following specific types of asbestos since there are important clues already suggesting unique pathologic mechanisms with chrysotile compared to amphibole. Such studies will need to include asbestos-like fibers such as erionite and nanofibers, which could significantly expand the potential public health impacts of environmental autoimmunity if such fibers induce similar immune dysfunction. Importantly, if there is an autoimmune component to asbestos-related lung diseases, specifically targeting the adaptive immune system may provide better therapeutic approaches for fibrotic processes, leading to far better health outcomes.

## Figures and Tables

**Table 1:** Description of mineral fibers discussed.

Fiber family	Fiber names	Chemistry	Location/use
Serpentine	Chrysotile	Mg_3_(Si_2_O_5_)(OH)_4_ (idealized), rolled sheets of Si oxide tetrahedra	Many commercial uses [7], Sumas Mtn [30]

Amphibole	Actinolite Amosite Anthophyllite Crocidolite Tremolite	Various Mg, Fe, Ca, and Na ions on double chains of silicon oxide tetrahedra	Igneous and metamorphic rock, many commercial uses [7, 31, 32]

Asbestiform	Winchite Richterite	Similar to amphibole, not specifically classified as asbestos	Similar to amphiboles, contaminant [8, 31]

Nanomaterials	Nanotubes Nanowires	Many metal formulations, formed into very long, thin chains or tubes	Synthetic, many commercial uses [13, 14]

Zeolite	Erionite	(Na_2_,K_2_,Ca)_2_Al_4_Si_14_O_36_·15H_2_O (idealized), chains of silicate “cages” or rings	Igneous rock: Turkey [11, 12]; S. Dakota [11, 12]

**Table 2:** Selected studies evaluating antinuclear antibodies (ANA) and rheumatoid factor (RF) among asbestos exposed subjects.

Study, year [reference]	Exposure context, fiber type	Exposed group			Comparison group			Associated w/radiologic changes
		*n*	ANA+	RF+	*n*	ANA+	RF+	
Pernis et al. 1965 [18]	Insulation workers, chrysotile	315	—	25%	103	—	14%	

Turner Warwick and Parkes 1970 [23]	Medical screening, mixed	80	28%	27%				Yes

Turner Warwick 1973 [33]	Medical screening, mixed	196	20%	11.7%	—	—	—	Yes

Turner Warwick 1973 [33]	Factory workers, unknown	252	7.5%	5.3%	—	—	—	Yes

Turner Warwick 1973 [33]	Naval personnel, mixed	334	8.4%	3.6%	—	—	—	Yes

Lange 1980 [29]	Textile workers, unknown	58	21%	—	19	0%	—	Yes

Toivanen et al. 1976 [34]	Asb. miners, anthophyllite	66	1.5%	10.7%	—	—	—	

Kagan et al. 1977 [35]	Subjects with asbestosis	26	7.7%	35%	45	0%	11%	

Haslam et al. 1978 [36]	Subjects with asbestosis	28	35.7%	17.9%	—	—	—	Yes

Huuskonen et al. 1978 [25]	Varied: asbestos sprayers, insulators, cement, quarry	169	11.8%	22.5%	504	11%	—	No

Lange 1980 [29]	Asbestos textile workers	242	21%	10%	181	9%	—	Yes

de Shazo et al. 1983 [26]	Asbestos cement workers	31	0%	0%	51	0%	—	No

Doll et al. 1983 [37]	Asbestos cement workers	144	15%	3%	–	–	—	No

Lange 1980 [29]	Asbestos workers	39	50%	—	9	0%	—	

Zerva et al. 1989 [24]	Whitewash, tremolite (amphibole)	109	14%	—	34	34%	—	Yes (pleural)

Tamura et al. 1993, Tamura et al. 1996 [22, 38]	Asbestos plant workers	220	15%	3.2%	—	—	—	Yes (interstitial)

Nigam et al. 1993 [19]	Asbestos factory milling	71	12%	1.4%	28	7%	0%	

Pfau et al. 2005 [20]	Contaminated vermiculite Amphiboles	70	70%	33%	50	40%	36%	Yes

**Table 3:** Animal model studies of asbestos and autoimmunity.

Reference	Strain (all inbred)	Disease model	Sex used	Treatment (fiber, route, duration)	Notes
Ferro et al., 2013 [39]	C57BL/6 mice	None	Female	LA, Chry, i.t., 7 mo.	LA (not Chry) increased ANA and IL-17
Pfau et al., 2008 [40]	C57BL/6 mice	None	Female	LA, i.t., 7 mo.	LA increased ANA, anti-Ro52, anti-dsDNA, IC
Salazar et al., 2012 [41]	Lewis rat	None	Female	LA, amosite, i.t., 13 weeks	Both increase ANA, anti-Jo-1. No IC, no anti-dsDNA
Salazar et al., 2012 [42]	Lewis rat	Antigen-induced arthritis (CIA, PG-PS)	Female	LA, amosite, i.t., 13 weeks	Both fibers increase ANA; no exacerbated disease
Pfau et al., 2011 [43]	C57BL/6 mice	None	Female	LA, tremolite, i.t., 7 mo.	Both induced antifibroblast antibodies

LA: Libby amphibole; ANA: antinuclear antibodies; Chry: chrysotile; i.t.: intratracheal; CIA: collage-induced arthritis; PG-PS: peptidoglycan/polysaccharide induced arthritis; IC: immune complexes in kidneys. Amosite and tremolite are both amphiboles.
